# Spatially-offset Raman spectroscopy for monitoring mineralization of bone tissue engineering scaffolds: feasibility study based on phantom samples

**DOI:** 10.1364/BOE.10.001678

**Published:** 2019-03-06

**Authors:** Max Dooley, Aruna Prasopthum, Zhiyu Liao, Faris Sinjab, Jane McLaren, Felicity R. A. J. Rose, Jing Yang, Ioan Notingher

**Affiliations:** 1 School of Physics and Astronomy, University of Nottingham, University Park, Nottingham NG7 2RD, UK; 2 School of Pharmacy, University of Nottingham, University Park, Nottingham NG7 2RD, UK

## Abstract

Using phantom samples, we investigated the feasibility of spatially-offset Raman spectroscopy (SORS) as a tool for monitoring non-invasively the mineralization of bone tissue engineering scaffold *in-vivo*. The phantom samples consisted of 3D-printed scaffolds of poly-caprolactone (PCL) and hydroxyapatite (HA) blends, with varying concentrations of HA, to mimic the mineralisation process. The scaffolds were covered by a 4 mm layer of skin to simulate the real *in-vivo* measurement conditions. At a concentration of HA approximately 1/3 that of bone (~0.6 g/cm^3^), the characteristic Raman band of HA (960 cm^−1^) was detectable when the PCL:HA layer was located at 4 mm depth within the scaffold (i.e. 8 mm below the skin surface). For the layers of the PCL:HA immediately under the skin (i.e. top of the scaffold), the detection limit of HA was 0.18 g/cm^3^, which is approximately one order of magnitude lower than that of bone. Similar results were also found for the phantoms simulating uniform and inward gradual mineralisation of the scaffold, indicating the suitability of SORS to detect early stages of mineralisation. Nevertheless, the results also show that the contribution of the materials surrounding the scaffold can be significant and methods for subtraction need to be investigated in the future. In conclusion, these results indicate that spatially-offset Raman spectroscopy is a promising technique for *in-vivo* longitudinal monitoring scaffold mineralization and bone re-growth.

## 1. Introduction

A common treatment for major bone damage is autologous bone grafting [1]. Autologous grafting is not the optimal solution because the quality and quantity of bone grafts that can be harvested is sometimes not sufficient to meet demand, it increases the risk of infection, and can lead to haemorrhaging, cosmetic disability, nerve damage, and a loss of function. An alternative to autologous grafts is the use of tissue engineered scaffolds that can be implanted in the defect to offer a 3D structure to support and stimulate the regeneration and repair of the bone [2]. *In vivo* models where scaffolds are implanted in critical bone defects in large animals (e.g. sheep) have been commonly used to model the healing process in humans [3]. These studies provide a better understanding of the bone repair process in order to optimise the physical and chemical properties of the material to reduce healing time and improve the quality of the newly formed bone. For a critical bone defect, the damage to the bone is so great that the body fails to repair the bone correctly [4]. What will constitute a critical defect depends on the size of the bone damaged [5,6], and the age and health of the patient [7,8]. When a defect reaches this critical size, the body fails to fill the defect with extracellular matrix (e.g. collagen) and to mineralise; instead, the exposed damaged bone is repaired leaving an indent or hole in the tissue with repaired sides, leading to bone tissue that is weaker than before the damage [9]. When using a scaffold, the quality of the repaired bone depends on the ability of cells to populate and remodel the scaffold. Thus, longitudinal data regarding mineral deposition within the scaffold is desirable and important for optimizing the physiochemical properties of the scaffold.

Typically, the quality of the repaired bone is evaluated by end-point histological tests. However, histology is destructive and therefore can be used only at the end time-point (typically 4-6 weeks after implantation). 3D micro-computed tomography (μCT) is commonly used to analyze the morphology and mineral density of newly formed bone in animal models [10], including for *in-vivo* longitudinal studies [11,12]. μCT has also been combined with single photon emission computed tomography (SPECT) in order to obtain more detailed molecular information during the course of bone formation and remodeling [13]. Although this technique requires radioactive SPECT probes, a study using synthetic hydrogel scaffolds implanted in critical size calvarial defects generated in mice, showed that *in-vivo* longitudinal data regarding morphology and bone density agreed with end-point histological and μCT evaluations [13].

Raman spectroscopy (RS) is a non-destructive spectroscopic technique that has high chemical specificity and does not require exogenous labels or probes [14]. RS has been widely used for the analysis of bone tissue [15–17] and bone tissue engineering scaffolds [18–22]. Spatially-offset Raman Spectroscopy (SORS) is a variant RS technique that is able to recover molecular information of bone *in-vivo* transcutaneously [23,24]. Recently, we demonstrated the feasibility of using SORS to measure Raman spectra of hydroxyapatite (HA) powder buried in layers of polymer and ceramic tissue engineering scaffolds as thick as few millimetres, covered by 1 mm thick skin layer [25]. While these feasibility studies indicated the potential of SORS for measuring *in-vivo* longitudinal data from small animal model studies, a better understanding of the SORS signals is required in order to optimise the instrumentation for *in-vivo* measurements (i.e. a hand-held probe) and support the data analysis. By increasing the maximum offset of the device and having control over the size of the collection points the sensitivity of the device was optimised.

Here we have developed a series of phantom samples to mimic the mineralisation of scaffolds implanted in a large animal critical bone defect and the measurement conditions for *in-vivo* longitudinal study. The samples consisted of 3D printed composite scaffolds with polycaprolactone (PCL) and hydroxyapatite (HA) microparticles, for which the concentration of HA varied to simulate different degrees of mineralisation [26]. The experiments were carried out using a table-top SORS instrument based on a digital micro-mirror device (DMD) [27], that allowed flexible adjustments of the spatial offsets in order to optimise the measurement conditions and develop the design of a future fibre-optics SORS probe that could be used in real animal studies.

## 2. Materials and methods

### 2.1 Spatially-offset Raman spectroscopy (SORS) instrument

The DMD-based SORS instrument was equipped with a 785 nm wavelength laser (Xtra II, Toptica). A 100 mm focal length 2-inch diameter lens was used to focus the laser beam on the sample (120 mW power, spot size ~0.5 mm) and to collect the backscattered Raman photons. After passing through a dichroic filter that blocked the elastically scattered photons, the Raman photons were focused with a lens on a software-controlled DMD (size 14.4 mm x 8.8 mm, resolution 1920 x 1080 pixels, DLP6500 Texas Instrument). As the DMD was located in a plane conjugate to the sample, it allowed the selection of multiple spatially offset collection points (0.22 mm size) distributed in a semi-circle around the point conjugated to the laser excitation, equivalent to spatial offset values in the range of 0-4 mm. The Raman photons reflected by the DMD collection points were analyzed by a spectrometer (Holospec, Andor) equipped with a deep-depletion back-illuminated CCD (iDus 420, Oxford Instruments). For a selected spatial offset, the SORS spectrum was calculated as the sum of the spectra corresponding to all DMD collection points on the corresponding semi-circle, after aligning and calibrating them along the wavenumber axis. For each spatial offset, 18 repeat spectra were measured at an acquisition time of 10 s per spectrum.

### 2.2 3D printing of PCL:HA scaffolds

**Fig. 1:**
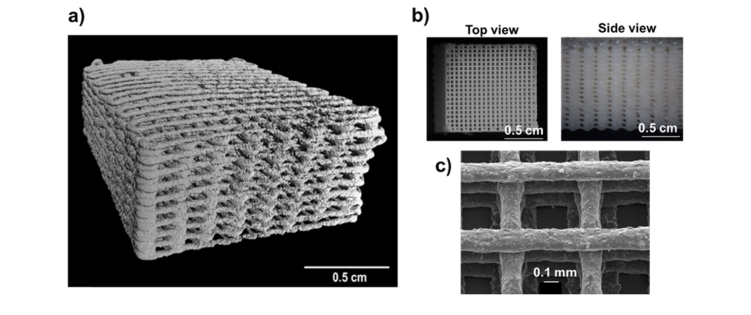
Representative images of the 3D printed PCL:HA scaffolds used in this study. a) μCT 3D reconstruction image of a PCL:HA 1:4 scaffold (1 x 1 x 0.5 cm^3^), porosity = 64.7%; b) Images from dissecting microscope of a PCL:HA (1:4) scaffold (1 x 1 x 1 cm^3^). c) Scanning electron microscopy of a PCL:HA scaffold with 1:2 blend ratio.

Porous scaffolds with the same pore size and different dimensions were designed by BioCAD software (Fig. 1

). A viscous polycaprolactone (PCL)/dichloromethane (DCM) solution was vigorously mixed with hydroxyapatite (Sigma Aldrich, UK) to create different ratios of PCL and HA in the order of 4:0, 4:1, 1:1, 1:2 and 1:4 (Table 1

**Table 1:** Scaffold and bone samples used in this study

Ratio PCL:HA	Percentage HA by Mass	HA density g/cm^3^ ( ± 0.01)
1:0	0%	0
4:1	20%	0.18
1:1	50%	0.68
1:2	67%	0.83
1:4	80%	0.93
Bone	~70% [28]	~2 [28]

).

The solution was loaded into a printing cartridge and directly deposited through a 25G tip (250 µm ID, Adhesive Dispensing, UK) using 3D Discovery printer (regenHU, Switzerland) with a pressure of 6 bar and a printing speed of 5 mm/s. Rapid DCM evaporation led to solidification of the printed construct. Table 1 converts these weight ratios into HA concentration to allow a better comparison with data available in the literature for typical mineralised bone [28].

### 2.3 Phantom samples

Figure 2

**Fig. 2:**
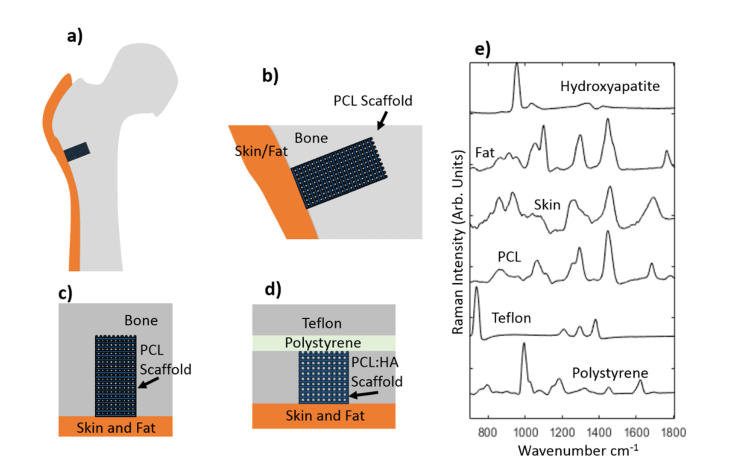
a) Diagram of the head of a femur with a critical defect drilled into it with the position of the scaffold shown. b) Close up of the defect filled with a PCL scaffold. c) A schematic of a defect in the same orientation as the phantom for comparison. d) A schematic of the phantom used in this study, designed to mimic a critical defect. e) Raman spectra of the materials that make up the phantom (spectra normalized between 0 and 1 and shifted vertically for clarity).

describes schematically the intended use of the scaffolds in large animal models and the phantom sample developed in this study to mimic the mineralisation process and the *in-vivo* SORS measurements. For real *in-vivo* experiments, the scaffold is implanted into a critical defect drilled to a diameter of 8 mm and 15 mm depth in the femur of a sheep and covered by a layer of skin (typically 3-5 mm thick) [29] (schematically described in Fig. 2(a)-(c)). The phantom consists of several parts made of different materials (Fig. 2(d)). Multiple 1.5 mm thick layers of Teflon were layered to create an 18 mm thick phantom with a square hole (10 mm x 10 mm x 10 mm) which mimicked the critical defect. Behind the Teflon slab, a 5 mm thick layer of polystyrene (PS) was placed in order to give an indication of whether the femur bone at the back of the defect would contribute to the measured SORS signal. After the insertion of the scaffolds (sizes 10 mm x 10 mm x 2mm) in the Teflon hole, the phantom sample was covered by a layer of pig skin (sourced from a local retail outlet) cut to 4 mm thickness, with a large enough surface area to cover the face of the phantom. While this phantom closely resembled the *in-vivo* measurement conditions, the materials used were selected to have similar light scattering properties to bone [2] but distinctive Raman bands to allow an understanding of the contributions of various regions of the phantom to the measured SORS spectra. This is particularly important for SORS measurements where different parts of the sample can be probed depending on the value of the spatial offset. Figure 2(e) presents the Raman spectra of each material used for the phantom. The spectrum of HA has a strong band at 960 cm^−1^ (phosphate band) [15–17]. The PCL has Raman bands assigned to the C-O-C vibrations as a triplet at 1067 cm^−1^, 1098 cm^−1^, and 1110 cm^−1^, and CH_2_ bands at 1300 cm^−1^ and 1445 cm^−1^ [4]. Teflon and PS have strong Raman bands at 734 cm^−1^ and 1004 cm^−1^ respectively. Therefore, the use of these two materials for the bone phantom effectively allows us to identify the contributions of the sides (Teflon) and back (PS) of the critical defect to the measured SORS spectrum without interfering with our measurement of the 960 cm^−1^ band from HA. Because the molecular composition of pig skin changes with depth, Raman spectra were measured from the top epidermis/dermis part (bands at 970 cm^−1^ and 1300 cm^−1^ assigned to collagen) and from the adipose tissue at the bottom part (strong 1368 cm^−1^ band) [30,31].

### 2.4. Data analysis

First, all spectra were normalised to minimum 0 and maximum 1. The difference between the SORS spectra of the PCL:HA scaffold and PCL-only scaffold (control sample) at each offset value was then calculated using an in-house iterative algorithm. To eliminate errors caused by small baseline or intensity variations (likely due to small differences in optical scattering properties between samples), a correction factor in the form of a 2nd order polynomial was included in the subtraction algorithm. This polynomial was determined by minimising the square difference between the two SORS spectra in an iterative method. The spectral region 930-980 cm^−1^ containing the main HA band at 960 cm^−1^ was excluded from the minimisation. A number of 30 iterations was used, as this was observed to lead to stable solutions in all cases.

## 3. Results and discussion

### 3.1 Testing the limits of detection for the PCL:HA scaffolds

**Fig. 3:**
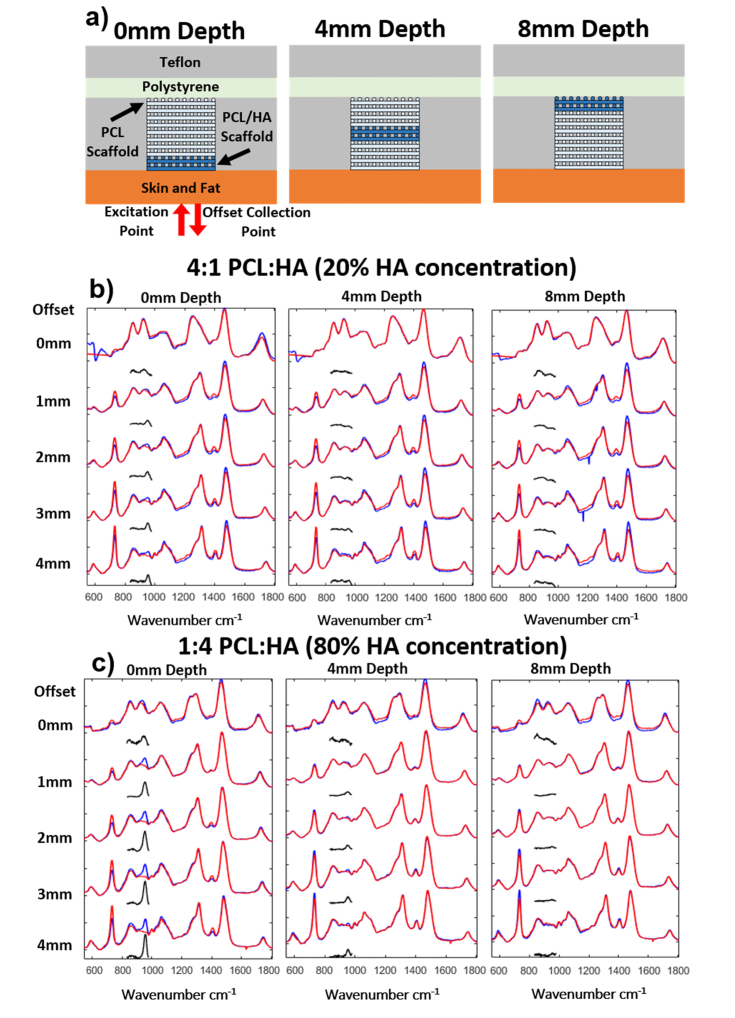
a) Schematic of the phantom with a single 2 mm thick PCL:HA scaffold layer at 0, 4 and 8 mm depth. b) SORS spectra for 4:1 PCL:HA ratio scaffolds at 0, 4, and 8mm depths. c) SORS spectra for 1:4 PCL:HA. The blue lines are the spectra of the phantoms containing the blended PCL:HA layers; the red line are for PCL only scaffolds (control). The black lines present the difference spectra blue minus red lines in the 830-1000 cm^−1^ region (2x magnified).

First, we tested the detection limit of the SORS measurements for PCL:HA layers with varying HA concentration, placed at different depths within the phantom sample. This was achieved by filling the defect hole with five 2 mm thick layers of scaffold, four of these were PCL-only and one layer of PCL:HA blend (Fig. 3a

). For each ratio of the PCL:HA blend, the PCL:HA layer was placed in turn at 0, 2, 4, 6, and 8 mm beneath the skin layer. A sixth sample containing five PCL-only scaffold layers was used to mimic the initial conditions of a newly implanted scaffold into the defect. For all samples, SORS spectra were acquired using spatial offsets of 0, 1, 2, 3 and 4 mm. Figure 3b,c present the SORS spectra for the phantoms containing PCL:HA layers with the lowest and highest concentrations of HA.

Figure 3b,c show that at 0 mm spatial offset, all SORS spectra were dominated by bands assigned to the top skin layer, in particular epidermis/dermis. Increasing the spatial offset to 1 mm already enabled the detection of Raman bands assigned to the deeper layers of the sample, including the detection of the 960 cm^−1^ band assigned to HA, as well as bands assigned to the adipose tissue in skin (1445 cm^−1^ band), PCL (bands in the 1000-1100 cm^−1^ region), and Teflon (734 cm^−1^). When the spatial offset was further increased (2-4mm range), the contribution of the subsurface layers of the phantom increased, allowing even detection of the polystyrene (PS) (1004 cm^−1^ band) back layer. For the PCL:HA 4:1 layer (HA concentration ~10 times lower than in bone), the HA band was detected only when the PCL:HA layer was at the top of the scaffold, immediately under the skin layer (i.e. 0 mm depth).

However, for the PCL:HA 1:4 layer (HA concentration roughly half that of bone), SORS was able to detect the 960 cm^−1^ band even when the layer was 4 mm deep in the scaffold. The presence in the SORS spectra of the 734 cm^−1^ band assigned to Teflon indicated that the side walls of the defect hole contributed to the measured SORS spectra. This result suggests that in an *in-vivo* experiment, the bone side walls of the defect would also contribute to the SORS spectra and the band at 960 cm^−1^ may overlap the signal from the mineralising scaffold. This contribution, which could be established at the start of the experiment (i.e. day zero), will have to be taken into account when attempting to determine the concentration of HA within the scaffolds.

**Fig. 4:**
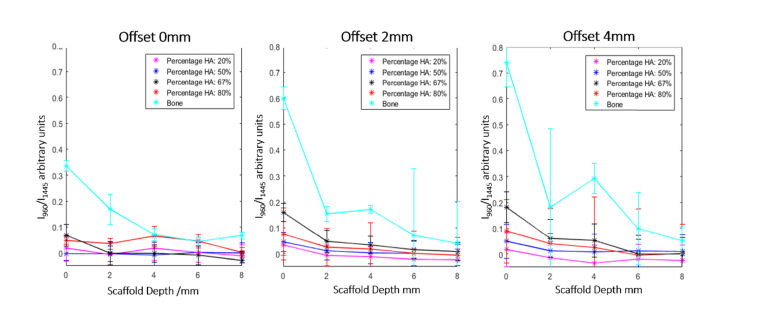
The ratio between the intensity of the 960 cm^−1^ band in the difference spectra and the 1445 cm^−1^ band in the spectra of the PCL:HA samples. Error bars represent the standard error of the mean for 18 repeat measurements.

In order to provide a quantitative measure of HA detection from the SORS spectra, the ratio between the 960 cm^−1^ band (HA) in the difference spectra and the 1445cm^−1^ band in the PCL:HA spectrum (CH_2_ deformations, mainly in the skin and PCL scaffold) was calculated for all samples analysed (Fig. 4

). The 1445 cm^−1^ band was selected as an internal standard based on the assumption that the adipose tissue in the skin would have the lowest variation compared to any other parts of the bone defect. For comparison, a set of measurements were also carried out using a 2 mm thick bone sample from the leg of a sheep (sourced from a local retail outlet). The results in Fig. 4 show that for 0 mm offset, the SORS spectra cannot discriminate between the different concentrations of HA present in the scaffolds. However, at 2 mm offset, the I_960_/I_1445_ ratio decreases as the PCL:HA layers are located deeper in the sample and as the concentration of HA decreases. The HA remained distinguishable until the PCL:HA layers were placed at 6 mm depth where the data started to overlap.

These results lead to the conclusion that the 4 mm spatial offset provides the highest detection sensitivity and resolution for detection of HA. The 4 mm offset was the largest offset tested in this investigation (limitated by the size of the DMD and instrument optics), and it is possible that larger offsets may have yielded higher sensitivity. The larger separation between the signals means that the larger offsets may be able to differentiate between smaller changes in mineralisation and therefore be more useful for longitudinal *in-vivo* measurements. The results indicate that the limit of detection for HA depends on the depth, ranging from 0.18 g/cm^3^ (~10x smaller than bone) at the top of the scaffold immediately under the skin layer to ~0.61 g/cm^3^ (~1/3 of bone concentration) at 4 mm depth in the scaffold. For the PCL:bone samples, the 960 cm^−1^ band was broader compared to the bands recorded for the PCL:HA scaffolds, likely due to the more complex molecular composition of bone (HA present in more crystalline phases). Because all spectra were analysed using the algorithm optimised for the PCL:HA scaffolds, the analysis led to some larger error bars for the PCL:bone samples at 4 mm offsets.

### 3.2 Phantoms for mimicking in-vivo mineralization patterns

Next, phantom samples were constructed to investigate the feasibility of SORS to monitor *in-vivo* biologically relevant models for bone repair, with the focus on scaffold mineralization. The aim was to determine the lowest levels of HA that can be detected and to investigate whether the SORS spectral and temporal profiles can discriminate between spatially homogenous and non-homogenous mineralization models.

#### 3.2.1 Uniform mineralization model

**Fig. 5:**
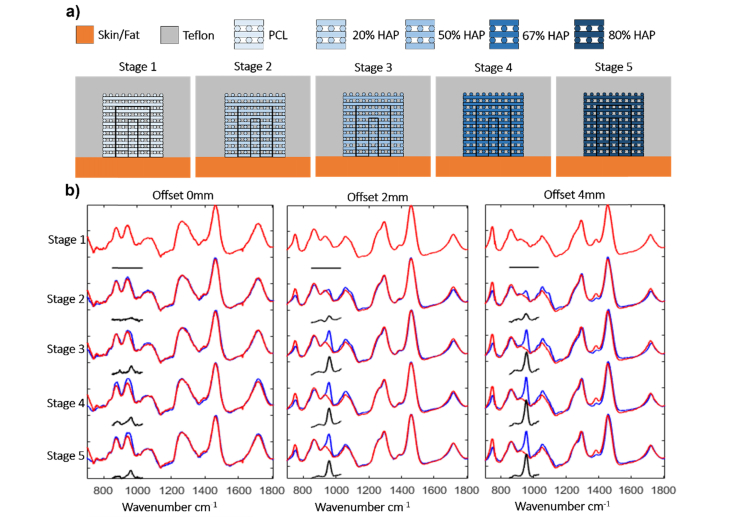
a) A schematic of the phantoms used for the uniform mineralization model. b) SORS spectra of the phantoms mimicking the uniform mineralisation model. Blue lines: SORS spectra of PCL:HA scaffolds, Red lines: SORS spectra of the PCL-only scaffold in the phantom; Black line the difference between the blue and red lines in the 830-1000 cm^−1^ range.

In this model the phantoms mimicked a process by which the mineralisation of the implanted PCL scaffold undergoes a uniform mineralisation throughout the PCL scaffold. The samples used in this test were PCL-only and PCL:HA scaffolds of 10 mm x 10m m x 10 mm inserted in the Teflon slab covered by the 4 mm pig skin layer (schematically presented in Fig. 5a

). The amount of HA in the PCL:HA scaffolds were increased using the ratios 4:1 - 1:4 in order to mimic different time steps in the process of mineralisation, from the moment of implantation to ~1/2 the HA concentration of mature bone.

Figure 5b presents the SORS spectra measured from the uniform PCL:HA scaffolds within the phantom sample at different spatial offsets. At 0 mm offset, the SORS spectra were dominated by Raman bands assigned to connective tissue in skin. The 960 cm^−1^ band assigned to HA was detected only for the PCL:HA scaffolds with rations higher than 1:1 (~0.29 g/cm^3^). Increasing the spatial offset to 2 mm allowed the detection of the 960 cm^−1^ band corresponding to HA in all scaffolds, even at the lowest HA concentration of 4:1 (0.18 g/cm^3^). For a given spatial offset (apart from 0 mm), the intensity of the 960 cm^−1^ band increases with the increased concentration of HA. The data also indicated that for a sample with a given PCL:HA ratio, increasing the spatial offset value from 2mm to 4 mm led to an increase in the intensity of the 960 cm^−1^ band (the Raman photons travelling or generated deeper in the scaffold contribute more to the spectra). The deeper sampling also led to an increase in the intensity of the 734 cm^−1^ band corresponding to Teflon (side walls of the defect). However, for a given spatial offset value, the contribution from the defect side walls (i.e. Teflon signal) was constant and could therefore be subtracted. Compared to the SORS spectra in Fig. 3, for a given spatial offset value and PCL:HA ratio, the spectra corresponding to the uniform PCL:HA scaffolds (Fig. 5) show a higher ratio between the intensity of the bands at 960 cm^−1^ assigned to HA and the 1090 cm^−1^ band corresponding to the PCL.

These results indicate that the combination between the I_960_/I_1090_ and I_960_/I_1445_ ratios could be used to quantify the density of the HA and the HA depth extend into the PCL scaffold.

#### 3.2.2 Gradual inward mineralization

**Fig. 6:**
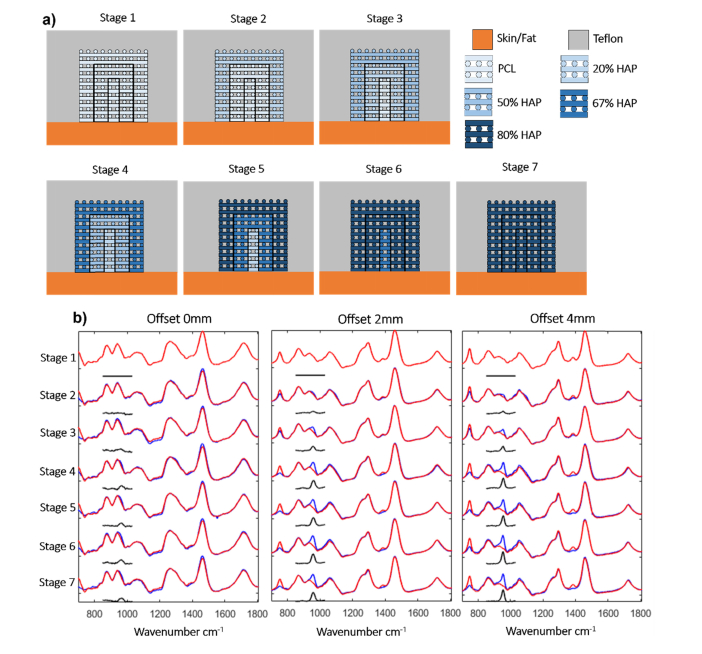
a) A schematic of the phantoms used for the gradual inward mineralization model. The 7 stages are shown with the shade of the layers of the phantom indicating the concentration of HA in the PCL/HA scaffold with a key of the colors used. c) SORS spectra for the graduated mineralisation model. The blue line is read with the blended material and the red line is the PCL only scaffold in the phantom. The black line is the difference between the two.

The second model studied here was the graduated inwards mineralisation of the scaffold. In this model, HA mineralisation of the extracellular matrix deposited within the scaffold starts at the surface of the old bone defect and grows into the scaffold with a concentration gradient of HA into the scaffold. We used seven different configurations of the phantom from all PCL-only to uniform 1:4 PCL:HA (Fig. 6a

). A naming convention was designed to describe the scaffolds in the defect: the name uses the ratio of concentration in each of the layers naming them from outside inwards so 41:PCL:PCL is the 4:1 PCL:HA blend on the outside with two layers of PCL inside. Figure 6 shows that, as with the previous samples, the Raman spectra recorded at 0 mm spatial offset had low sensitivity for HA as the main contribution was from the top skin layer. At increased offsets, the 960 cm^−1^ band was detected in all spectra, even at the lowest concentration of HA such as the 41:PCL:PCL. This sample represents low levels of the mineralisation starting from the surface of the defect, as only the exterior of the scaffold contains HA at a density of 0.18 g/cm^3^.

**Fig. 7:**
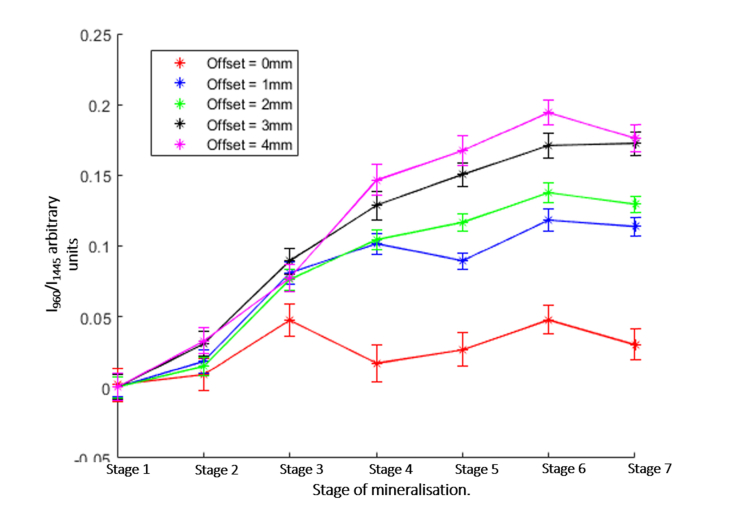
Analysis of the SORS spectra for the gradual inward mineralization model. The ratio between the intensity of the 960 cm^−1^ band in the difference spectra (PCL:HA minus PCL-only scaffolds) and the 1445cm^−1^ band in the spectra of the sample containing HA. Each stage from 1 to 7 represents an increase in HA concentration. Error bars represent the standard error of the mean for 18 repeat measurements.

These results indicate that for this model, HA present in the scaffold is detectable by SORS even at concentrations an order of magnitude lower than the concentration of complete mineralisation of bone. Increasing the offset to 4 mm provided a similar increase in the intensity of the 960 cm^−1^ band, but at the same time, an increase in the signal from the Teflon layers surrounding the scaffold. Therefore, the use of 2 mm offset seemed to be more advantageous as less background signal from the side walls of the bone defect are detected. In real measurements, this background signal can overlap the signal from the newly deposited HA in the scaffold. To evaluate the optimal conditions for quantification of HA in the phantom samples, Fig. 7

presents the calculated ratio between the intensity of the 960 cm^−1^ band in the difference spectra (PCL:HA minus PCL-only scaffolds) and the 1445cm^−1^ band in the spectra of the sample containing HA (I_960_/I_1445_). The results show that the relative intensity of the 960 cm^−1^ band increased when the concentration of HA and the spatial offset increased. The highest sensitivity and detection resolution for HA was observed when using the 4 mm spatial offset. However, once the outer PCA:HA layer of the scaffold reached the 1:4 ratio, the I_960_/I_1445_ ratio seemed to plateau, regardless of the value of the spatial offset (higher than 0), indicating a decrease in detection resolution for HA.

## 4. Conclusions

The use of tissue engineering scaffolds for stimulating bone re-growth in critical bone defects is a promising way to improve bone healing; however, monitoring bone growth *in situ* remains a challenge. Using phantom samples based on 3D-printed PCL:HA scaffolds we investigated the feasibility of SORS to monitor mineralisation of bone tissue engineering scaffolds in large animal models. The tests investigating the detection limits for mineralisation showed that SORS is able to detect HA concentrations at an order of magnitude lower than that found in living bone, even through a 4 mm thick layer of skin (mimicking *in-vivo* transcutaneous measurements). These low concentrations are only detected when the HA was located immediately under the skin surface. As the HA concentration increased so did the depth at which the HA was detectable. For the highest concentration of HA, the detection depth increased to 4 mm. Bone has a higher concentration of HA than any of the scaffolds investigated here and a 2 mm thick layer produced a stronger signal across all depths as seen in Fig. 4. The I_960_/I_1445_ band ratio can be a useful parameter to attempt longitudinal quantification of HA concentration, but would require a means to measure the thickness of the skin layer, which can vary during the 4-6 week duration of the real *in-vivo* experiments. Nevertheless, such changes in skin thickness may be measured using complementary techniques, such as ultrasound imaging.

For the experiments mimicking the uniform scaffold mineralisation, setting a spatial offset larger than 2 mm allowed sensitive detection of HA. Similar results were observed for the inward gradual mineralisation model. Nevertheless, the highest sensitivity and detection resolution for HA was observed when using the 4 mm spatial offset (largest offset possible with current instrument), which indicates that even the earliest mineralisation stage included in this study was detectable (i.e. only the outer 2 mm layer of the scaffold contained HA at a concentration ~10x lower than bone). The results also show that the surrounding walls of the bone defect also contributed to the measured SORS spectra (Teflon signal), that may overlap the Raman bands from the HA in the scaffold. For this reason, it would be advantageous to start the acquisition of SORS spectra as soon as the scaffold was implanted in order to establish a baseline SORS spectrum and quantify the changes in the HA signals during the 4-6 week period of bone re-growth. The ability to follow the bone healing process on the same animal will provide higher quality data with ethical and economic benefits from reducing the number of animals used during the research.
